# Percutaneous vertebroplasty versus kyphoplasty for the treatment of neurologically intact osteoporotic Kümmell’s disease

**DOI:** 10.1186/s12893-021-01057-x

**Published:** 2021-01-29

**Authors:** Shou-qian Dai, Rong-qing Qin, Xiu Shi, Hui-lin Yang

**Affiliations:** 1 Department of Orthopaedics, The First Affiliated Hospital of Soochow University, 188 Shizi St, Suzhou, 215006 Jiangsu China; 2 Department of Emergency Medicine, The First Affiliated Hospital of Soochow University, 188 Shizi St, Suzhou, 215006 Jiangsu China; 3 Department of Spinal Surgery, Gaoyou Hospital Affiliated Soochow University, 116 Fuqian St, Gaoyou, 225600 Jiangsu China; 4 Department of Orthopedics, Gaoyou People’s Hospital, 116 Fuqian St, Gaoyou, 225600 Jiangsu China; 5 Department of Obstetrics and Gynecology, The First Affiliated Hospital of Soochow University, 188 Shizi St, Suzhou, 215006 Jiangsu China

**Keywords:** Kümmell’s disease, Percutaneous vertebroplasty, Percutaneous kyphoplasty, Osteoporosis, Vertebral compression fracture

## Abstract

**Background:**

Percutaneous vertebroplasty (PVP) and kyphoplasty (PKP) have been widely used to treat neurologically intact osteoporotic Kümmell’s disease (KD), but it is still unclear which treatment is more advantageous. Our study aimed to compare and investigate the safety and clinical efficacy of PVP and PKP in the treatment of KD.

**Methods:**

The relevant data that 64 patients of neurologically intact osteoporotic KD receiving PVP (30 patients) or PKP (34 patients) were analyzed. Surgical time, operation costs, intraoperative blood loss, volume of bone cement injection, and fluoroscopy times were compared. Occurrence of cement leakage, transient fever and re-fracture were recorded. Universal indicators of visual analogue scale (VAS) and Oswestry disability index (ODI) were evaluated separately before surgery and at 1 day, 6 months, 1 year, 2 years and the final follow-up after operation. The height of anterior edge of the affected vertebra and the Cobb’s angle were assessed by imaging.

**Results:**

All patients were followed up for at least 24 months. The volume of bone cement injection, intraoperative blood loss, occurrence of bone cement leakage, transient fever and re-fracture between two groups showed no significant difference. The surgical time, the operation cost and fluoroscopy times of the PKP group was significantly higher than that of the PVP group. The post-operative VAS, ODI scores, the height of the anterior edge of the injured vertebrae and kyphosis deformity were significantly improved in both groups compared with the pre-operation. The improvement of vertebral height and kyphosis deformity in PKP group was significantly better than that in the PVP group at every same time point during the follow-up periods, but the VAS and ODI scores between the two groups showed no significant difference.

**Conclusion:**

PVP and PKP can both significantly alleviate the pain of patients with KD and obtain good clinical efficacy and safety. By contrast, PKP can achieve better imaging height and kyphosis correction, while PVP has the advantages of shorter operation time, less radiation volume and operation cost.

## Background

Kümmell’s disease (KD), also known as intravertebral avascular osteonecrosis, is a special type of osteoporotic vertebral compression fracture (OVCF) that most often occurs in the thoracolumbar segment of the spine [1]. The main clinical manifestations are: varying degrees of spinal trauma several weeks or months ago, delayed vertebral collapse and progressive deformity of kyphosis, causing low back pain [2]. KD can be divided into three types according to clinical and imaging findings: Type I back pain or no symptom with body height loss less than 20% and no adjacent disc degenerative disease (DDD); type II back pain with/without radiculopathy with body height loss more than 20% and adjacent DDD; and type III back pain with/without cord injury and posterior cortex breakage with cord compression [3, 4].

At present, most scholars have basically reached a consensus on the treatment of type III KD [5], but there are still controversies about the choice of surgical methods for type I or II KD (also called neurologically intact osteoporotic KD). Minimally invasive percutaneous vertebroplasty (PVP) and percutaneous kyphoplasty (PKP) both have good clinical effect in treating neurologically intact osteoporotic KD [6–8]. However, it is still inconclusive which surgical method has more advantages.

Therefore, a prospective study of type I or II KD treated with PVP or PKP in our hospitals from January 2016 to January 2018 was conducted to compare and explore their safety and clinical efficacy, and to provide references for clinicians to choose precise treatment plans.

## Methods

### Patient population

**Fig. 1:**
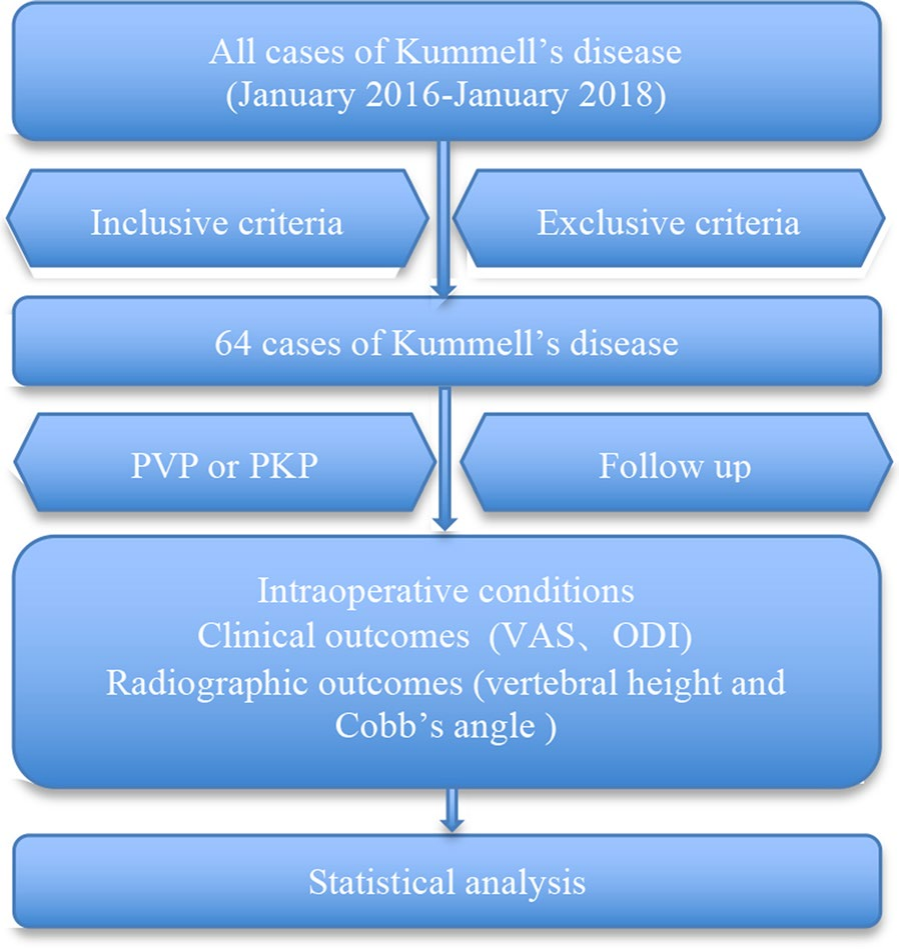
Cases of Kümmell’s disease and follow-up period

After the written approval of Institutional Review Board, 64 cases (30 males and 34 females) with neurologically intact osteoporotic KD from January 2016 to January 2018 were analyzed in this study; 30 underwent PVP and 34 underwent PKP. The baseline data of all patients and the follow-up period are shown in Table 1 and Fig. 1. All included cases must underwent plain films, computed tomography (CT) scan and magnetic resonance imaging (MRI). Preoperative dual-energy X-ray confirmed that all cases can be diagnosed as osteoporosis. All patients with clinical data and pictures gave written informed consent. Clinical and imaging data have obtained the written consent of all patients.

**Table 1:** Comparison of baseline data between two groups

Parameter	PVP	PKP	t/χ^2^	*P*
Case	30	34		
Sex				
Male (cases)	9	12	0.203	0.653
Female (cases)	21	22		
Age (years old)	75.81 ± 7.12	75.12 ± 6.92	0.393	0.696
BMI (kg/m^2^)	23.37 ± 3.52	23.15 ± 3.44	0.355	0.724
BMD (T value)	− 3.67 ± 0.66	− 3.62 ± 0.73	0.286	0.776
Course of disease (months)	7.22 ± 2.77	7.65 ± 2.61	0.639	0.525
Mean follow-up time (months)	28.78 ± 8.33	27.16 ± 7.67	0.810	0.421
Fall history				
Yes (cases)	11	10	0.617	0.537
No (cases)	19	24

The PVP group contained 30 cases (9 males, 21 females), aged 60–89 years old (average 75.81 ± 7.12 years old). The body mass index (BMI) was 19.45–28.82 kg/m^2^ (average 23.37 ± 3.52 kg/m^2^). The course of disease lasted 3–15 months (average 7.22 ± 2.77 months). The BMD T value was − 2.69 to − 5.63 (average − 3.67 ± 0.66). No obvious injury history was found in 19 patients and the remaining 11 patients had a history of falling before hospitalization. All cases were KD with single-segment lesion located at the following thoracic (T) or lumbar (L) vertebrae: T8 (2 cases), T9 (1 case), T10 (3 cases), T11 (4 cases), T12 (11 cases), L1 (6 cases), L2 (2 cases), and L3 (1 case) (Table 1).

The PKP group contained 34 cases (12 males, 22 females), aged 59–90 years old (average 75.12 ± 6.92 years old). The BMI was 19.11–29.27 kg/m^2^ (average 23.15 ± 3.44 kg/m^2^). The course of disease lasted 4–16 months (average 7.65 ± 2.61 months). The BMD T value was − 2.62 to − 5.98 (average − 3.62 ± 0.73). No obvious injury history was found in 24 patients and the remaining 10 patients had a history of falling before hospitalization. All cases were KD with single-segment lesion located at the following thoracic (T) or lumbar (L) vertebrae: T9 (2 cases), T10 (2 cases), T11 (6 cases), T12 (8 cases), L1 (10 cases), L2 (4 cases), L3 (1 case), and L4 (1 case) (Table 1).

There was no significant difference in gender, age, BMI, BMD, disease courses, affected segment of the two groups (all *P* > 0.05, Table 1).

### Inclusive and exclusive criteria

#### Inclusive criteria

(1) Patients with single-segment KD; (2) PVP or PKP was conducted bilaterally; (3) The intravertebral cleft (IVC) existed in CT images; (4) MRI confirmed the existence of low signal intensity on T1-weighted images and a low or high signal intensity on T2-weighted images, depending on whether the gas or fluid fills the cleft [9]; (5) Patients with BMD T value less than − 2.5; (6) Follow up for more than 2 years.

#### Exclusive criteria

(1) Patients with neurological deficit; (2) Patients had a history of spinal surgery; (3) Patient with malignant tumors, coagulopathy, or mental disorder; (4) Patients with pathological fractures; (5) Patients with signs of inflammation or infection at the puncture site; (6) Patients with severe cardiopulmonary dysfunction who were unable to tolerate the surgery procedure.

### Surgical procedures

Following the same principle, the same group of physicians performed PVP and PKP operation under stringent sterile conditions. The patients were placed in a prone position with abdomen vacant for position reduction. Bilateral pedicles were punctured separately under fluoroscopic guidance.

#### PVP procedure

Both lower extremities were elevated and pulled backward by two assistants, while head side traction via the patient's axilla and shoulder was performed by another assistant. The operator pressed the back of the patient to maintain a maximum hyperextension position. After successful positioning, the working sleeve reached the IVC area in the anterior-middle 1/3 junction of the involved vertebra. Then polymethylmethacrylate (PMMA, Tecres SpA, Verona, Italy) was carefully injected into the crack using a bone cement injector until the whole IVC region was fully filled and the cement was well diffused.

#### PKP procedure

PKP operation was performed according to the standard procedure introduced by Yang et al. [10]. Under biplanar fluoroscopic guidance, a transpedicular approach was used with cannula systems. The balloons (Kyphon, Sunnyvale, CA, USA) were carefully inserted through the cannulas and reached the anterior 3/4 of the injured vertebrae from a lateral view. Then the balloons were inflated slowly to minimize the risk of vertebral fracture and to create a cavity for the PMMA cement. The bone cement was immediately injected into the IVC region when it became doughy and could stand at the tip of the bone cement inserter. The whole injection process was strictly monitored under fluoroscopic control in the lateral plane.

### Clinical and radiographic evaluation

Clinical efficacy: bone cement leakage in the operation, intraoperative blood loss, volume of bone cement injection, and surgical time of the two groups were recorded (Table 2). The incidence of complication such as acute pulmonary embolism, nerve damage, transient fever and re-fracture were also recorded (Table 3). The pain degree of low back was assessed by VAS score [11] and the severity of the dysfunction was assessed by ODI score [12].

**Table 2:** Comparison of intraoperative conditions between two groups

Parameter	PVP	PKP	t/χ^2^	*P*
Surgical time (min)	32.34 ± 6.87	42.41 ± 7.32	5.652	0.000
Operation cost (CNY)	16,523.41 ± 2363.46	33,706.87 ± 2508.94	28.090	0.000
Volume of bone cement injection (mL)	4.30 ± 0.88	4.68 ± 0.91	1.693	0.096
Intraoperative blood loss (mL)	16.23 ± 4.45	17.27 ± 4.92	0.882	0.381
Fluoroscopy times	14.86 ± 4.03	20.54 ± 4.68	5.168	0.000
Bone cement leakage in the operation (n/%)	5/16.67	4/11.76	0.317	0.574

**Table 3:** Incidence of complications in the two groups

Complication	PVP (n = 30)	PKP (n = 34)
Acute pulmonary embolism	0	0
Nerve damage	0	0
Occurrence of cement leakage	5	4
Transient fever	2	3
Re-fracture	3	2

Radiographic evaluation: the height of the anterior edge of the injured vertebrae and the kyphotic angle on the lateral radiograph were recorded before and after the operation [13].

### Statistical analysis

Results were presented as mean ± standard deviation. Statistical analyses were implemented by SPSS 19 (SPSS Inc., Chicago, IL, USA). Independent sample t test was performed to compare the age, BMI, BMD, course of disease, mean follow-up time, surgical time, operation cost, volume of bone cement injection, intraoperative blood loss, fluoroscopy times, VAS, ODI, and imaging index between the PVP and PKP groups. Chi-squared test was used to compare gender, fall history and bone cement leakage. One-way ANOVA was performed to compare VAS, ODI, vertebral height and the Cobb’s angle in the same group. *P* value of less than 0.05 was considered statistically significant.

## Results

### Intraoperative conditions and complications

All 64 patients successfully completed the operation according to the preoperative plan. No serious complications such as acute pulmonary embolism, nerve injury or spinal cord compression occurred during the operation (Table 3). The operation time, operation cost and the intraoperative fluoroscopy times in PKP group were significantly higher than those in PVP group (*P* < 0.05). The volume of bone cement injection, intraoperative blood loss and bone cement leakage of the two groups showed no significant difference (*P* > 0.05) (Table 2).

9 cases (14.1%) of the patients had bone cement leakage during the operation, most of which were leakage along the fracture line. But no clinical symptoms occurred in the two groups. In other cases, the bone cement was well dispersed in the middle or anterior position of the vertebral body. During the postoperative rehabilitation, transient fever appeared in 2 patients (6.7%) in the PKP group and 2 patients (5.9%) in the PKP group. Body temperature returned to be normal after supportive treatment. During the postoperative follow-up period, 3 patients (10.0%) in the PVP group and 1 patient (2.9%) in the PKP group had re-fractures (Table 3).

### Clinical evaluations

**Fig. 2:**
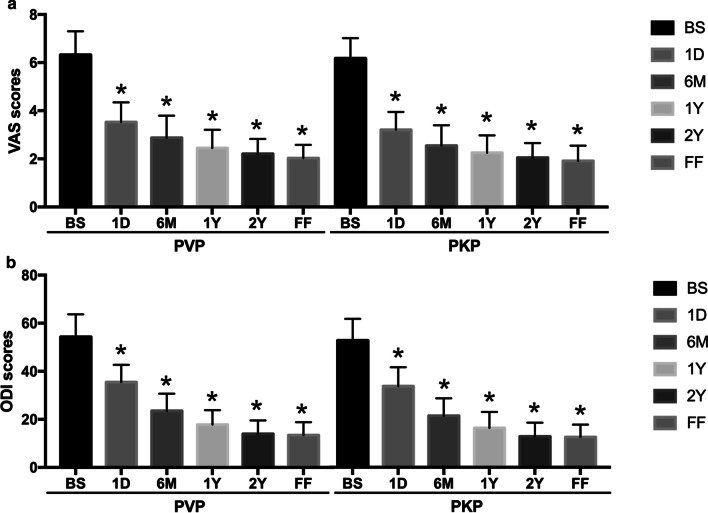
Comparison of VAS scores and ODI before and after surgery in the two groups. All patients were followed up for at least 22 months. Compared with before surgery (BS), both VAS (**a**) and ODI (**b**) scores of the two groups during the follow-up period [1 day (1D), 6 months (6 M), 1 year (1Y), 2 years (2Y), and final follow up (FF)] were shown significantly lower. However, there was no significant difference of both scores between PVP and PKP groups at each time point postoperatively. *P < 0.05 vs BS in each group

All patients were followed up for at least 22 months. No significant difference was found in the follow-up period between PVP and PKP groups (*P* > 0.05). Compared with before operation, both VAS and ODI scores of the two groups during the follow-up period (1 day, 6 months, 1 year, 2 years, and final follow up) were shown significantly lower (*P* < 0.05). However, there was no significant difference of both scores between PVP and PKP groups at each time point postoperatively (*P* > 0.05) (Fig. 2).

### Radiographic outcomes

**Fig. 3:**
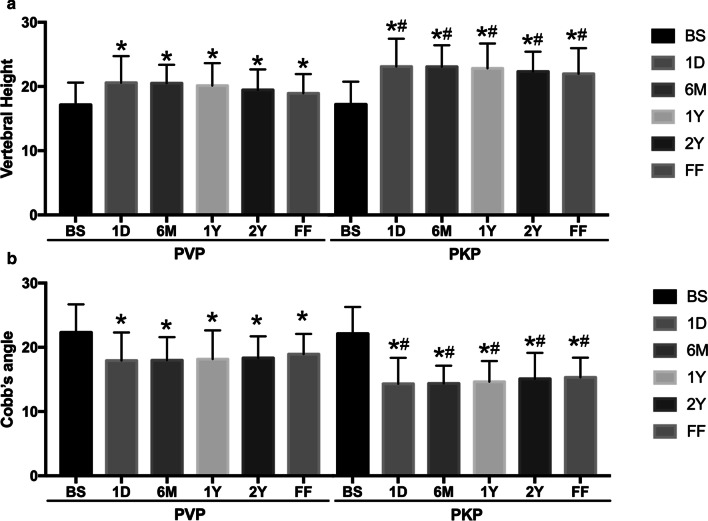
Comparison of the height of the anterior edge of the injured vertebral body and the Cobb’s angle before and after surgery in the two groups. Compared with before surgery (BS), two groups of height of the anterior edge of the injured vertebrae (**a**) and Cobb’s angle (**b**) at 1 day (1D), 6 months (6 M), 1 year (1Y), 2 years (2Y), and final follow up (FF) after operation were all statistically improved. The correction of injured vertebral height and kyphotic deformity in the PKP group was more obvious than that of PVP group at each time point. Even though correction of injured vertebral height and Cobb’s angle in both groups gradually decreased with time after operation, there were no statistically differences between 1 day and the final follow-up after operation in each group. *P < 0.05 vs BS in each group and ^#^P < 0.05 vs the same time point in PVP group

**Fig. 4:**
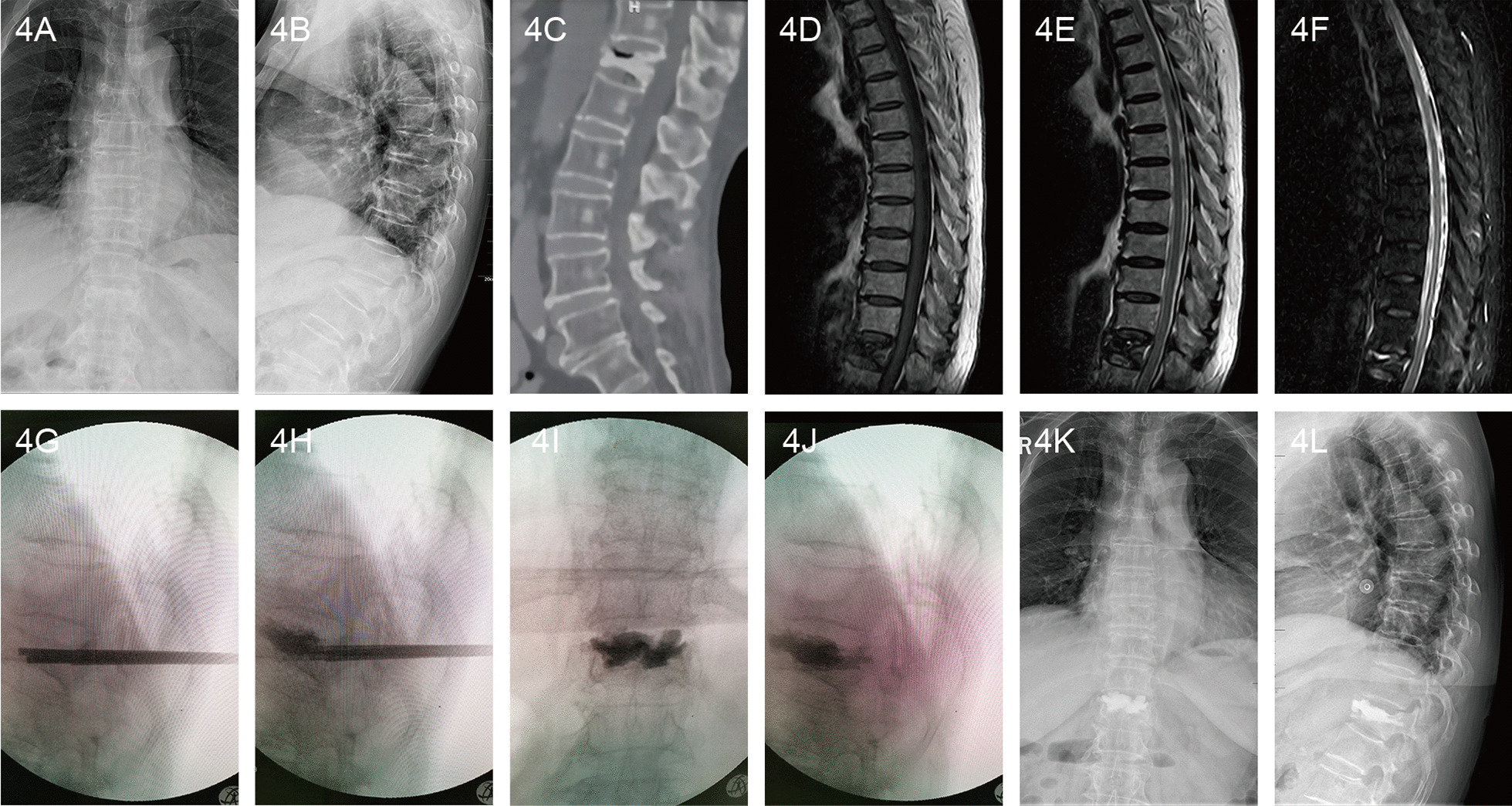
A 72-year-old female patient with Kümmell’s disease at T12 in PVP group: **a** Anteroposterior X-ray film before operation; **b** Lateral X-ray film before operation; **c** Intravertebral vacuum sign shown in sagittal CT image before operation; **d** A low signal intensity in the location of the cleft shown in sagittal T1-weighted MRI image before operation; **e**, **f** A well-defined low signal intensity in the location of the cleft shown in sagittal T2-weighted and short tau inversion recovery(STIR) MRI image before operation; **g** The height of the injured vertebral body was partly recovered after position reduction; **h** The PMMA diffused into the intervertebral trabecular space; **i**, **j** The bone cement filled the cleft without leakage in X-ray immediately after operation; **k** Anteroposterior X-ray film after operation; **l** Lateral X-ray film after operation

**Fig. 5:**
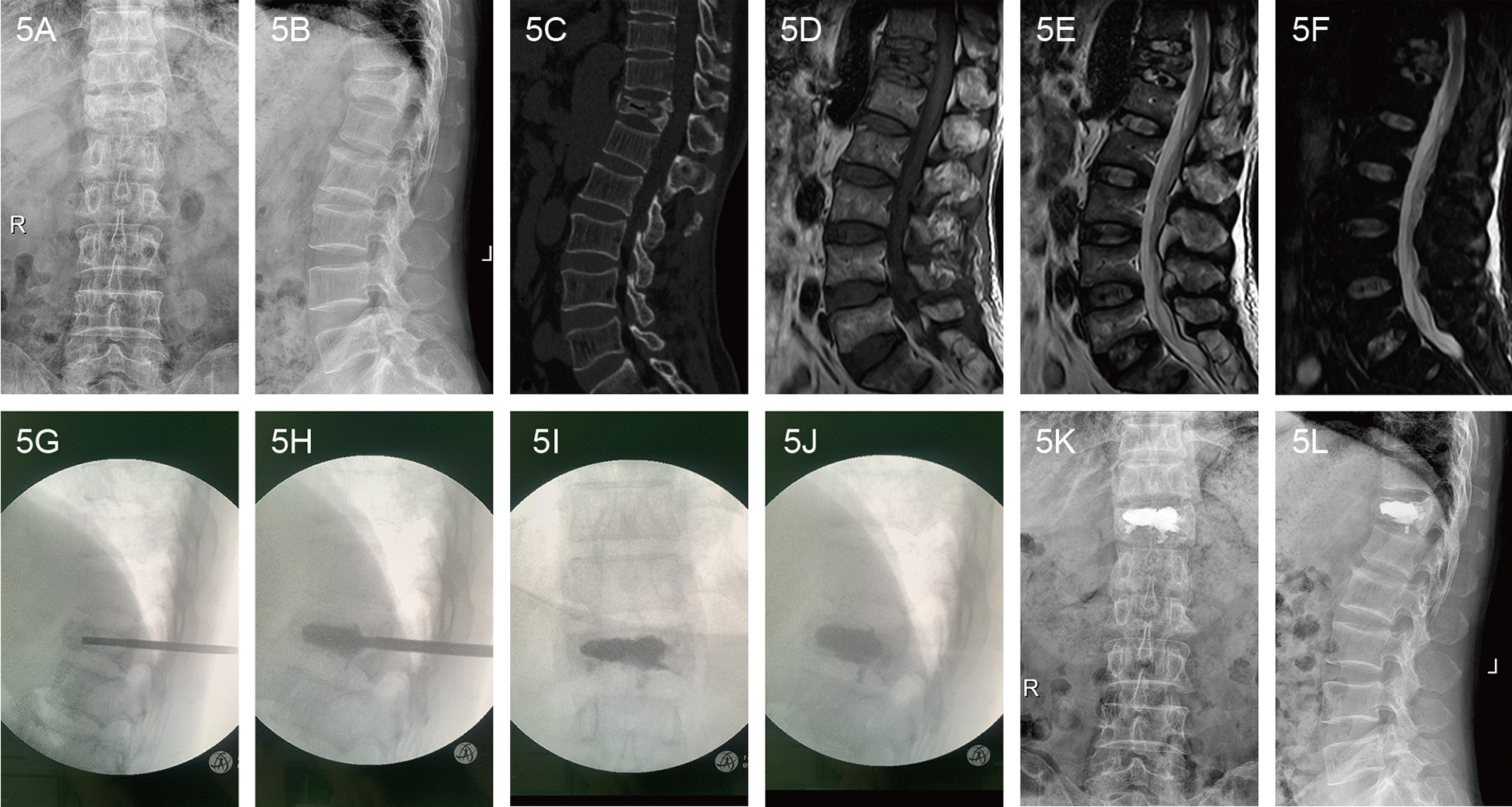
A 69-year-old male patient with Kümmell’s disease at T12 in PKP group: **a** Anteroposterior X-ray film before operation; **b** Lateral X-ray film before operation; **c** Intravertebral vacuum sign shown in sagittal CT image before operation; **d** A low signal intensity in the location of the cleft shown in sagittal T1-weighted MRI image before operation; **e**, **f** A well-defined low signal intensity in the location of the cleft shown in sagittal T2-weighted and short tau inversion recovery(STIR) MRI image before operation; **g** The height of the injured vertebral body was partly recovered after position reduction; **h** The balloons were inserted and placed inside the anterior 3/4 of the vertebral body from a lateral view, creating a cavity for the injected cement; **i**, **j** The bone cement filled the cleft without leakage in X-ray immediately after operation; **k** Anteroposterior X-ray film after operation; **l** Lateral X-ray film after operation

Compared with the pre-operation, two groups of height of the anterior edge of the injured vertebrae and Cobb’s angle at 1 day, 6 months and the final follow-up after operation were all statistically improved (*P* < 0.05). What’s more, the correction of injured vertebral height and kyphotic deformity in the PKP group was more obvious than that of PVP group at each time point (*P* < 0.05). Even though correction of injured vertebral height and Cobb’s angle in both groups gradually decreased with time after operation, there were no statistically differences between 1 day and the final follow-up after operation in each group (*P* > 0.05). More detailed data of patients are show in Fig. 3 and typical cases were shown in Figs. 4 and 5.

## Discussion

The clinical features of Kummell’s disease (KD) include: a history of trauma, asymptomatic period after trauma, delayed vertebral collapse and the existence of the vacuum or fissure signs in the injured vertebral body. It is reported that the pathological mechanism of KD is complicated and may be the result of the interaction of a variety of factors such as avascular necrosis in the vertebral body, changes in the biomechanics of the spine, and incomplete repair of bone microfractures [14]. The majority of researchers believes that the disease starts from osteoporotic vertebral fractures, followed by avascular necrosis of the injured vertebrae and pseudojoint formation, eventually leading to vertebral body collapse. The treatment of KD mainly includes conservative treatment, minimally invasive PVP or PKP and open reduction internal fixation [15]. Most of KD is still treated with surgery at present. PKP and PVP have been widely used in patients with neurologically intact osteoporotic KD, especially those who are intolerant to general anesthesia [16–18]. So far, many studies have reported the good effects of PVP and PKP in the treatment of KD [19–21], but it is still controversial that which surgical method is more advantageous. On the basis of these researches, we initiated a comparative study on patients with type I or II KD treated with PVP and PKP.

Our data showed that the operation time, operation cost, and intraoperative fluoroscopy times in the PVP group were statistically lower than those in the PKP group (*P* < 0.05). The volume of bone cement injection in the PKP group was higher than that in the PVP group, and the incidence of postoperative bone cement leakage in the PVP was higher than that in the PKP group, however, these differences were not statistically significant. Many previous similar studies have also confirmed that the rate of bone cement leakage in PVP was higher than that of PKP [22–24]. The possible explanation is: in PKP, the surgeon creates a low pressure environment before injecting bone cement into the injured vertebrae, which could reduce the penetration of bone cement to surroundings, compared with the high pressure environment of PVP. Meanwhile, during the balloon expansion of the PKP, the surrounding bone can be compressed more tightly, further sealing the fracture and reducing leakage. Our team has carried out a number of researches on PKP since 1999 and gradually formed a set of bone injection technique that can effectively reduce bone cement leakage, which was called temperature gradient cement injection technique. The key technical points include: (1) Choosing the best initial injection time point for bone cement; (2) Following the principle of injection slowly with low pressure; (3) Temperature gradient injection technique (i.e. interval injection technology); (4) Bone cement layered modulation technique if necessary. Both temperature gradient injection technique and bone cement layered modulation technique are applied in our cases. The insignificant statistical results obtained in this study may be related to the insufficient sample size or the different tendency to choose PVP and PKP. Since the PKP group is mostly patients with poor preoperative postural reduction, we will continue to expand sample size in order to draw more accurate conclusion. The serious surgical complications of PKP and PVP also include pulmonary embolism, nerve injury, and delayed cement displacement [25]. Fortunately, there was no serious complications in our study. We believed that antiosteoporotic treatment and rehabilitative exercise of the muscle strength of the waist and back are helpful to prevent delayed displacement. In addition, regular and timely follow-up after operation is also necessary. Regarding choice of surgical methods for type I or II KD, we prefer the safer PKP to reduce or avoid surgical risk.

Past studies have found that whether it is unilateral or bilateral injection of bone cement, the axial vertebral rigidity and strength can both be restored, and that the injection of bone cement into the pedicle on both sides can achieve good biomechanical performance [26]. Even so, in order to achieve consistency in the treatment of KD patients, we only selected bilateral puncture cases. It was reported that PKP is superior to PVP in treating KD with regard to improvement of vertebral heights and kyphosis deformity [27, 28]. In our study, compared with before operation, two groups of height of the anterior edge of the injured vertebrae and Cobb’s angle at 1 day, 6 months and the final follow-up after operation were significantly corrected (*P* < 0.05). The improvement of injured vertebral height and kyphotic deformity in the PKP group was significantly better than that of PVP group at each time point. At the last follow-up, there was no significant loss in the vertebral height and the Cobb’s angle of the kyphosis did not change significantly. Similar results were also confirmed by Zhang’s study [27] that PKP can obtain more satisfactory reduction results in the treatment of KD. According to Zhang et al. [29], the correction of Cobb’s angle in the PKP group was slightly better than the PVP group and there were no significant differences between two groups. The reason for the different results may be the different sample size and follow-up time in the two groups. The sample size and follow-up period in our study was larger and more easier to find the difference, and our conclusion was therefore more convincing. Our finds were also verified by a systematic review and meta-analysis that PKP is superior to PVP in terms of vertebral height recovery and correction of kyphosis [30]. The reason that PKP is better than PVP in correcting the height and kyphotic deformity of the injured vertebral body may be: PKP can increase the space for bone cement implantation through the mechanical expansion of the balloon in the injured vertebrae. At the same time, the balloon can directly restore the vertebral height and correct the kyphotic deformity, which is not available in PVP. The comparison of imaging data in our research indicated that the injected bone cement all reached or exceeded the midline of the injured vertebrae, and well dispersed in the middle or anterior position of the vertebral body. In addition, there are cavities and fissures in the vertebral body of KD patients, and the compression and collapse of the vertebral body mainly occur in the anterior or middle vertebral column, which also accorded with the biomechanical characteristics of the spine [31].

The purpose of surgical treatment of KD is not only to obtain better imaging results, but also to relieve pain and restore function as soon as possible. For some elderly patients with or without severe osteoporosis, it is not even necessary to excessively restore the vertebral height and Cobb’s angle. More than 2 years of follow-up found that VAS and ODI scores of both PVP and PKP groups were significantly lower at 1 days, 6 months, 1 year, 2 years and the final-up after surgery than before surgery, but no significant difference between PVP and PKP groups was found at all time points postoperatively. This indicated that PVP and PKP have basically the same curative effect in the treatment of type I or II KD and both surgical methods can significantly relieve pain and improve the life quality of KD patients. We believe that the main reason of bone cement injection for pain relief may be that bone cement fills the IVC of the injured vertebrae and plays the role of physical support. Similar conclusions was also made by some studies which believed that both PKP and PVP operation can obviously relieve pains and have little difference in improving the postoperative function of patients [29, 32, 33]. Considering the safety and the better reduction of PKP, we suggest that for patients with no financial concerns, PKP treatment should be given priority on the basis of informed consent to patients and their families.

However, there are some important limitations of this study. The sample size is small and non-RCT articles might induce various types of bias. To confirm our findings, a large multi-center randomized RCT should be conducted. In addition, duration of follow-up period in both groups varied and might bring negative impact on our results.

## Conclusions

PVP and PKP can both significantly alleviate the pain of patients with KD and obtain good clinical efficacy and safety. By contrast, PKP can achieve better imaging height and kyphosis correction, while PVP has the advantages of shorter operation time, less radiation volume and operation cost.

## Data Availability

The datasets used during the current study available from the corresponding author on reasonable request.

## Abbreviations

KD: Kümmell’s disease
VAS: Visual analogue scale
ODI: Oswestry disability index
BMD: Bone mineral density
OVCF: Osteoporotic vertebral compression fracture
IVC: Intravertebral cleft
PKP: Percutaneous kyphoplasty
PVP: Percutaneous vertebroplasty
